# A low-cost easily implementable physiotherapy intervention clinically improves gait implying better adaptation to lower limb prosthesis: a randomized clinical trial

**DOI:** 10.1038/s41598-021-00686-9

**Published:** 2021-10-27

**Authors:** Leticia Vargas Almeida, Claudiane Arakaki Fukuchi, Tania Emi Sakanaka, Alberto Cliquet

**Affiliations:** 1 Department of Orthopedics and Traumatology, Faculty of Medical Sciences, State University of Campinas, São Paulo, Brazil; 2 Biocybernetics and Rehabilitation Engineering Lab., Department of Electrical Engineering, University of São Paulo, São Paulo, Brazil

**Keywords:** Trauma, Rehabilitation

## Abstract

Lower limb amputation highly impacts the lives of individuals. The inability to walk due to difficulties in adapting to wearing prosthesis can potentially result in physical degeneration and comorbidity in this population. In this randomized clinical trial study, we investigated if a low-cost and easily implementable physiotherapy intervention was effective in improving gait performance and adaptation to lower limb prosthesis in individuals with an amputation. A total of 26 individuals participated in the study, 16 with lower limb amputation and 10 without amputation. Participants with amputation were further divided in intervention and control groups. The intervention group underwent a rehabilitation protocol aimed at strengthening muscles and improving prosthesis adaptation. Muscle strengthening targeted the hip segment, prioritizing the abdominal muscles, hip flexors, extensors, adductors and abductors, followed by cicatricial mobilization and weight-bearing on the stump for desensitization. Assessment and measures were performed across the kinetic and kinematic parameters of gait. In the comparison between pre-and post-intervention, a significant increase in gait speed (0.68—2.98, 95% CI, 1.83, effect size ES) and cadence (0.56—2.69, 95% CI, 1.63, ES) was found between groups and time points. Step (0.73—3.11, 95% CI, 1.92, ES) and stride length (0.62—2.84, 95% CI, 1.73) increased between pre- and post-intervention, while in the control group both variables remained smaller. The intervention group decreased stance phase as a percentage of gait cycle between pre- and post-intervention (− 1.33—0.62, 95% CI, − 36, ES), while it increased in the control group. Improvement in a combination of important gait parameters indicates that the intervention protocol promoted the adaptation to prosthesis and the functional independence of individuals with lower limb amputation. It is recommended that the participants continue receiving follow-up assessments and rehabilitation interventions.

## Introduction

Lower limb amputation (LLA) highly impacts the lives of individuals. Psychological, physical and social well-being are directly affected. Care centers for individuals with amputation, composed of multidisciplinary rehabilitation teams, are responsible for the recovery and functional maintenance of patients, as well as for their reintegration to work, sports and social activities^1^. One important factor that influences the quality of life of this population is adequate adaptation to prosthesis. Despite not being the only contributor, the inability to walk due to difficulties in adapting to wearing prosthesis can potentially result in physical degeneration and comorbidity in this population^2,3^. Inadequate adaptation to prosthesis is also considered one of the factors related to the high incidence of falls and fear of falling among them^4^, and should therefore be one of the focuses of rehabilitation programs directed at this population. Some studies report that confidence and balance during walking and the execution of specific tasks are factors that benefit the quality of life and socialization of these individuals, a prevalent dependency found in the lives of individuals with LLA^5–7^.

According to a systematic review about physiotherapy rehabilitation programs for individuals with amputations wearing prosthesis, there is no consensus regarding the types and quality of exercises and evaluations more suitable for this population. In general, the studies show the importance of strengthening exercises as well as gait training for prosthesis use. However, only 9 out of 10,393 studies met the inclusion criteria of this systematic review, and no clear conclusions were obtained on how to better assist this population with rehabilitation interventions^8^.

Furthermore, more clinical quantitative and qualitative assessments are needed to better understand the performance of gait in these individuals when wearing prosthesis following intervention. Among the different types of assessment, temporal-spatial, kinetic and kinematic analyses offer a quantitative approach that can precisely detect differences in performance^9–12^.

The objective of this study was to develop a simple and low-cost rehabilitation protocol, taking into account the wide-spread use of a functionally poor type of prosthesis offered by rehabilitation units from the public sector of many countries, and verifying the potential of applying this protocol in those units with few resources. The intervention prioritized the strengthening of the hip segment and gait training with the objective of stabilizing the pelvic girdle, optimizing gait performance and attaining good adaptation to prosthesis. It was elaborated and adjusted to last for the same amount of time reserved by the public hospital for prosthesis adaptation, 4 months in total. The purpose was to meet the functional needs of these individuals after amputation. The justification of the study is based on the difficulty in finding rehabilitation protocols in the literature that meet the primary needs of individuals with lower limb amputations that rely on care offered by public sectors with few resources, to verify if assistance could be satisfactorily provided without the need for expensive equipment, often also more difficult to manipulate. Temporal-spatial, kinetic and kinematic analyses were performed pre- and post-intervention.

## Method

### Design

This study is a randomized clinical trial registered with the Brazilian Clinical Trials Registration Identifier RBR-4s5nkh with the registration date 20/05/2020 (https://ensaiosclinicos.gov.br/metis/search/query), conducted in accordance with the Declaration of Helsinki guidelines, and approved by the State University of Campinas Research Ethics Committee (Nº. 2.349.249). Written informed consent was obtained from each participant before the study began.

### Participants

Data were collected with a total of 26 participants, including 16 participants with amputation and 10 able-bodied individuals. The clinical population, including the intervention and control groups, was recruited from the Specialization and Assistance Center for Individuals with Amputations at the Clinics Hospital. Sample selection was based on the following inclusion criteria: age between 18 and 60 years, physically inactive, intellectually capable, having undergone unilateral vascular or traumatic amputation at the transtibial (TT) or transfemoral (TF) levels, suitable for prosthesis. Able-bodied individuals were recruited from the hospital staff if met with the inclusion criteria: age between 18 and 60 years, physically inactive, intellectually capable, having no amputations or musculoskeletal injuries related to the locomotor system. Able-bodied individuals were submitted to the same kinetic and kinematic gait assessment performed by the groups of individuals with amputation. Its evaluation aimed to ensure that a ‘standard’ gait parameter was attained from able-bodied individuals undergoing this study’s experimental setup and procedure, comparable to the normative data already found in the literature, in view of the large variability found from one instrument to another.

Initial screening was performed from medical records, followed by interviews. All interviewed individuals with amputation agreed to take part in the study and were blindly randomized during recruitment. An independent researcher divided the sample into control and intervention groups by handling them blindly chosen envelopes containing the assigned group. The intervention group underwent the rehabilitation described below (Table 1).

**Table 1:** Prosthesis model and intervention protocol pre-and post-prosthesis.

Transtibial	Transfemoral
**Prostheses model**	
Endoskeleton in aluminum, with PTB/KBM/PTS support, with patella fitting and articulated sach foot, manufactured by Otto Bock	Endoskeleton in aluminum, monocentric knee, suspension with suction valve, articulated sach foot, quadrilateral socket with ischial support, manufactured by Otto Bock
**Intervention protocol**	
Pre-prosthesis (24 sessions)	Post-prosthesis (8 sessions)
Cryotherapy; Ice applied for 1 min on the entire distal surface of the stump	In orthostatic position, alternation of full weight bearing between limbs while holding parallel bars for 5 min
Desensitization of stump tissue; Brushing for 1 min with soft sponge and 1 min with rough sponge for desensitization	Simulation of walk-in-place, taking a step forward and returning to the initial position, with one limb and then with the other, while holding parallel bars
Stretching of ischio-tibialis and hip flexor muscles and execution of metabolic movements (dorsiflexion and plantar flexion) with elastic band (TheraBand®), 2 series of 12 repetitions each	Hip and knee flexion in place, one leg followed by the other, while holding parallel bars
In dorsal decubitus, abdominal muscles (rectus, obliques and transverse) strengthening exercises; 3 series with repetitions of 10, 12 and 14 using the ball	Sit-to-stand performed on benches with different heights, progressing from highest to lowest height bench
In dorsal decubitus, concentric and eccentric hip flexor muscles strengthening exercise; 3 series of 10, 12 and 14 repetitions using a band loop with increasing resistance	Forwards, backwards and sideways walk while holding parallel bars
In ventral decubitus, concentric and eccentric hip extensor muscles strengthening exercises; 3 series of 10, 12 and 14 repetitions using a band loop with increasing resistance	Forwards, backwards and sideways independent walk
In dorsal decubitus, concentric and eccentric hip adductor muscles strengthening exercises; 3 series of 10, 12 and 14 repetitions using a ball	Dual-task practice, upper limb activities added to walking with increasingly challenging obstacles
In lateral decubitus, concentric and eccentric hip abductor muscles strengthening exercises; 3 series of 10, 12 and 14 repetitions using a band loop	Dual-task practice, balancing on a mini-trampoline with increasingly challenging upper limb activities. Activities with a ball being thrown in different directions
In orthostatic position, stump full weight bearing exercise while stepping on different types of surfaces and holding parallel bars	Neuromuscular circuit training with cones where the patient performs independent forwards, backwards and sideways walk, gradually increasing speed of execution of gait, and gradually increasing speed and execution of circuit training
During all rehabilitation sessions, active-assisted muscle strengthening exercise was performed with electro stimulation (Russian current) of gluteus, for 20 min. With parameters modulated frequency 25 kHz, frequency 50 Hz, duty cycle 50%, time of contraction and rest of 3 s and ramps of ascent and descent off	Exercises going up and down steps and stairs, with progression, with and without handrail support
	Activities and progressions of gait training in the outdoor environment, on irregular terrain with ramps and stairs, simulating daily life conditions

### Intervention

The intervention protocol was designed to be low-cost and easy to apply. The use of accessible materials, such as ice, sponges, elastic resistance bands and band loops (TheraBand), balls, cones, benches, mini-trampolines, parallel bars, electrotherapy (Model: ENDOPHASYS MMS-050, Manufacturer / distributor: KLD Biosistemas Equipamentos Eletrônicos Ltda, Amparo-SP, Brazil), and the outdoor environment, were chosen so that public rehabilitation centers, with few resources, could use it as a first step in post-amputation care. The intervention protocol aimed at promoting adaptation to prosthesis use and functional and social independence. The exercises focused on core exercises, predominantly in the abdominal region, and on the activation of hip muscles^13,14^. The protocol was designed to meet capacity and demand needs, i.e., juggle between developing the necessary physical capacity and adapting to wearing prosthesis, ultimately minimizing the high physical demand required for this adaptation^15^.

The intervention group underwent the rehabilitation exercise protocol, divided in two phases: pre- and post-prosthesis. The pre-prosthesis phase was carried out for 12 weeks, with 2 sessions of 1 h each week, totalling 24 sessions. The post-prosthesis phase was carried out for 4 weeks, with 2 sessions of 1 h each week, totalling 8 sessions. The intervention period was set for 4 months in accordance with the time frame that the hospital normally takes to perform prosthesis preparations and adjustments. The prostheses were created according to the model provided by the public health service (Table 1). During the post-prosthesis period, adjustments and alignment of prostheses were performed by specialized technical professionals to comply with the participant comfort and needs.

### Outcome measures

Data used to characterize the sample was collected and filled in an evaluation sheet containing the items: sex, height, weight, age, body mass index (BMI), type of amputation, cause of amputation, and amputation time. Type, cause, and amputation time were obtained from the participants’ medical records.

**Figure 1:**
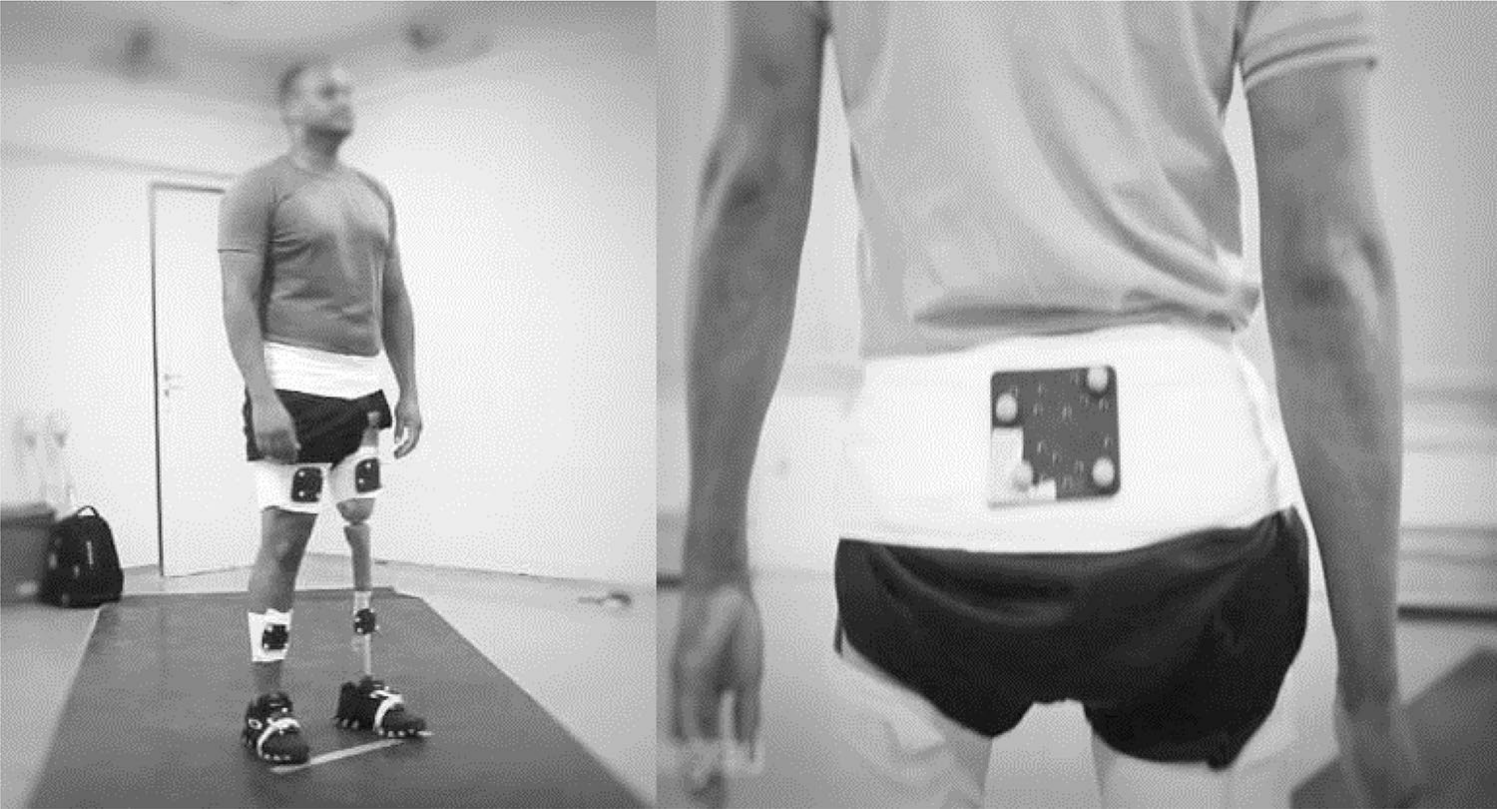
Positioning of clusters with retro-reflective marks fixed on the rigid plate.

The gait cycle evaluation protocol, including kinetic and kinematic evaluations, was performed by asking the participant to walk along an 8-m-long walkway, at a self-selected gait speed considered comfortable by each individual. Three valid attempts from each participant in all groups were recorded. Twelve high- speed cameras operating at 100 Hz (Vero 1.3, Vicon Motion Systems Ltd., Oxford, United Kingdom), and 2 force platforms at 1000 Hz (AMTI, Watertown, United States) were used to capture kinematic and kinetic data, respectively. The kinematic model used was a six degrees-of-freedom model created with the Motion Monitor X Gen software (v. 2019, Innsport Inc., Chicago Illinois, USA). Marker positions from a static trial were used to define joint centres. Calibration of the participant body segments were performed with clusters with 4 retro-reflective marks fixed on a rigid plate and placed over the lumbar-sacral region, thighs, legs and dorsal surface of the feet (Fig. 1). Kinetic and kinematic data were processed and analyzed with the Motion Monitor X Gen software. The phases of the gait cycle were defined as: initial contact, loading response, mid stance, terminal stance, pre-swing, initial swing, mid swing and terminal swing. Data from amputated and contralateral limbs were analysed in participants with amputation, but only data from the dominant limb in able-bodied participants were used for analysis. Data were analysed from the hips, knees and bilateral ankle joints in the sagittal plane. The temporal-spatial data of each group were also analysed. Data was filtered with a 4th order, zero-lag low-pass Butterworth filter with 10 Hz cut-off frequency. Gait analysis was performed in all individuals with amputation, after the pre-prosthesis intervention phase was completed by the intervention group and 12 weeks after recruitment of the control group. Two assessments were recorded, as soon as the participant received the prosthesis and 4 weeks after (post gait training for the intervention group). The group of able-bodied participants was tested once.

### Statistical analysis

**Figure 2:**
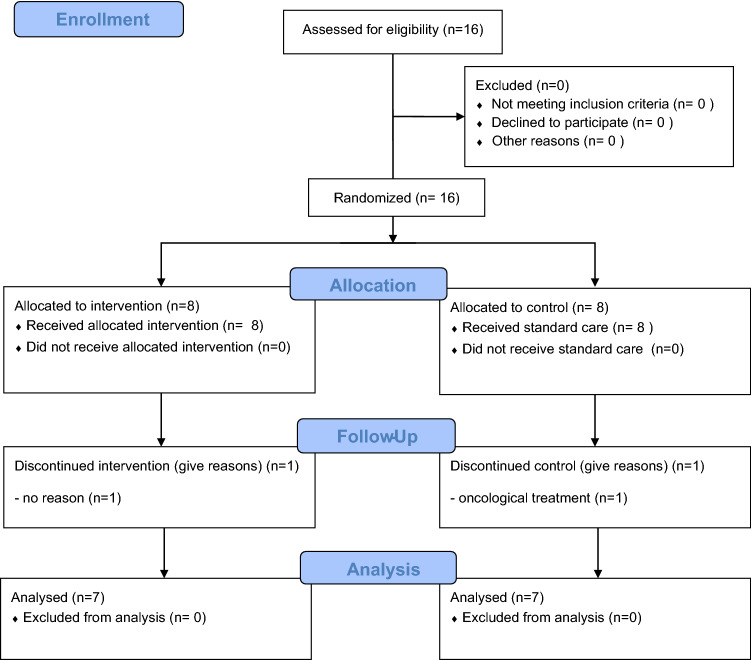
CONSORT flow chart (CONSORT, Consolidated Standards of Reporting Trials).

All statistical analyses were conducted with PASW Statistics software (v. 18.0, SPSS Inc., Chicago, USA). Initially we performed a characterization of the sample by amputation level (transtibial or transfemoral) and for able-bodied individuals. Participants with amputation were then further characterized in control and intervention groups (Fig. 2). Data distribution and homogeneity of variance were verified with Shapiro–Wilk and Levene’s test. For the comparison of continuous variables between groups of individuals with transtibial amputation, transfemoral amputation and able-bodied individuals, one-way repeated measures ANOVA, followed by Tukey post hot test was used when data presented normal distribution and homogeneity of variances; Kruskal Wallis test, followed by Bonferroni post hot test, was used when the data did not show normal distribution or homogeneity of variances.

For the comparison of the amputation time (pre- *versus* post-intervention) and amputation cause between TT and TF amputation groups, the Mann Whitney U-test and the Fisher exact test were used, respectively. The analyses were conducted independently between amputated and contralateral limbs. For the comparison of the sample characterization parameters between control and intervention groups pre-prosthesis, Student's t-test were used for continuous variables and Fisher's exact test for categorical variables. For the analysis of angle, moment and temporal-spatial variables, Mixed Linear Models were applied, assuming group (intervention x control), limb (amputated x contralateral) and time (pre x post-intervention) as fixed effects, and subject as a random effect. Additionally, time and limb effects were assumed as repeated measures and the injury level as covariable. When major effects or significant interactions were found, Sidak's post hoc test was used to point out the differences.

Due to the large number of possible comparisons, interactions were analyzed from largest (triple) to smallest (main effect) complexity, and only significant results composed by the largest number of factors were described in the final results. Additionally, the clinical effects of the intervention were further analyzed by calculating the effect size (ES, d Cohen), defined as the mean difference divided by the combined standard deviation (DPpooled) from pre- vs. post-intervention results, and interpreted as: small (│d│ ≤ 0.30), moderate (0.31 < │d│ < 0.8) and large (│d│ ≥ 0.8)^16^. Thereafter, to minimize the bias caused by the small sample size, a Hedges correction was applied to obtain ES and estimates of unbiased confidence limits^17^. A 5% (P ≤ 0.05) significance criterion was adopted.

## Results

### Characterization of the sample

There was no significant difference between groups of participants with TT and TF amputation and able-bodied adults with regards to age, height, body mass and body mass index. Moreover, no difference was found in amputation time and amputation cause between individuals with amputation groups. For the intervention and control groups at the start of the experiment before intervention, no statistical difference was found for age, height, body mass index, amputation time, cause of amputation and type of amputation. Although no significant difference was observed between groups in the sample characterization variables, it was decided to include the amputation type as a covariable in the subsequent analyses to minimize possible bias caused by the imbalance in the proportion of participants with TF and TT amputation in intervention and control groups (Table 2).

**Table 2:** Characterization of the total sample, divided by transtibial vs transfemoral amputation vs able-bodied group and by control vs intervention groups.

Variables	Groups			P-value	Groups		P-value
	Transtibial (N = 6)	Transfemoral (N = 8)	Able-bodied (N = 10)		Control (N = 7)	Intervention (N = 7)	
Age (years)	38 ± 13	40 ± 11	35 ± 13	0.655^AN^	37 ± 13	42 ± 9	0.478^TT^
Height (m)	1.72 ± 0.07	1.74 ± 0.11	1.76 ± 0.04	0.583^AN^	1.69 ± 0.08	1.77 ± 0.09	0.092^TT^
Body mass (kg)	75.9 ± 9.4	77.0 ± 12.7	84.5 ± 9.9	0.224^AN^	75.1 ± 9.6	78.0 ± 12.5	0.642^TT^
Body mass index (kg/m^2^)	25.7 ± 2.6	25.2 ± 1.9	27.3 ± 4.1	0.345^AN^	26.2 ± 1.7	24.6 ± 2.3	0.180^TT^
Amputation time (months)	12 (7 a 59)	11 (6 a 69)	–	0.896^ MW^	12 (6 a 120)	12 (8 a 24)	0.737^TT^
**Amputation cause [Nº. (%)]**							
Traumatic	4 (66.7)	7 (87.5)	–	0.538^EF^	6 (85.7)	5 (71.4)	1.000^EF^
Vascular	2 (33.3)	1 (12.5)			1 (14.3)	2 (28.6)	
Type of amputation [Nº. (%)]^c^							
Transfemoral	–	–	–	–	3 (42.9)	5 (71.4)	0.280^EF^
Transtibial	–	–	–	–	4 (57.1)	2 (28.6)	

Data are presented as mean ± standard deviation, median (1st to 3rd quartile) or frequencies of occurrence [No. (%)]. AN: One-way ANOVA; MW: Mann Whitney U-test; FE: Fisher's exact test. TT: Student's t-test; ^a^ Data presented as mean ± standard deviation; ^b^ Data presented as median (1st to 3rd quartile). ^c^ Data presented as absolute (N^o^.) and relative (%) frequencies.

### Kinematics: control vs intervention

Between amputated and contralateral limbs, it was observed differences in the ankle joint, knee joint and hip joint. Between groups of individuals with amputation, it was observed higher plantar flexion in the intervention group during initial contact compared to the control group (Table 3).

**Table 3:** Ankle, knee, and hip joint angles (º) in the sagittal plane during different phases of gait cycle, in amputees pre- and post-intervention.

Variables		Control		Intervention	
		Amputated limb	Contralateral limb	Amputated limb	contralateral limb
IC Ankle^G; L^	Pre	1.8 ± 2.7	− 4.6 ± 3.6^‡^	− 1.8 ± 2.2*	− 4.3 ± 3.5*^‡^
	Post	0.5 ± 5.4	− 0.7 ± 4.7^‡^	− 1.4 ± 3.3*	− 4.0 ± 4.9*^‡^
IC Knee^L^	Pre	3.1 ± 5.9	5.1 ± 3.7^‡^	1.7 ± 1.5	6.5 ± 6.0^‡^
	Post	3.7 ± 5.0	5.4 ± 8.2^‡^	1.3 ± 0.8	4.9 ± 5.4^‡^
IC Hip^G; T^	Pre	13.8 ± 6.6	11.8 ± 5.7	16.3 ± 8.9*	22.2 ± 5.5*
	Post	17.6 ± 4.0	18.2 ± 9.2^‡†^	17.2 ± 3.1*^†^	22.1 ± 6.5*^†^
LR Ankle^L^	Pre	− 5.3 ± 5.3	− 2.9 ± 3.0^‡^	− 7.3 ± 4.8	− 6.2 ± 4.8^‡^
	Post	− 5.8 ± 4.3	− 4.1 ± 2.8^‡^	− 6.6 ± 6.2	− 5.6 ± 4.4^‡^
LR Knee	Pre	6.7 ± 5.6	8.2 ± 3.6	5.2 ± 7.5	10.8 ± 7.8
	Post	4.4 ± 4.1	13.1 ± 4.2	5.9 ± 6.5	12.1 ± 5.0
LR Hip	Pre	9.1 ± 3.6	10.4 ± 7.1	9.9 ± 4.2	16.9 ± 3.7
	Post	9.5 ± 4.7	15.3 ± 11.5	13.3 ± 1.8	13.9 ± 6.3
MS Ankle	Pre	2.8 ± 2.0	2.7 ± 2.2	4.0 ± 3.1	4.2 ± 3.2
	Post	2.6 ± 2.3	2.2 ± 1.4	3.0 ± 3.5	3.8 ± 2.8
MS Knee	Pre	4.0 ± 3.9	3.9 ± 5.8	4.3 ± 7.0	6.7 ± 5.1
	Post	3.9 ± 4.5	7.9 ± 5.2	5.3 ± 4.9	5.0 ± 5.3
MS Hip^G^*^L^*^T^	Pre	2.0 ± 2.0	2.2 ± 4.4	2.3 ± 4.3	7.3 ± 4.3^‡^
	Post	4.2 ± 2.3	5.5 ± 6.8	4.4 ± 2.6^†^	1.3 ± 3.8^†^
TS Ankle^L^	Pre	2.5 ± 2.2	6.0 ± 2.9^‡^	3.4 ± 3.4	8.6 ± 5.3^‡^
	Post	5.0 ± 4.0	5.6 ± 6.3^‡^	4.7 ± 2.9	11.5 ± 4.7^‡^
TS Knee^G^*^L; L^*^T^	Pre	3.1 ± 5.0	3.0 ± 3.0	2.1 ± 7.3	5.6 ± 3.4^‡^*
	Post	3.8 ± 4.1	3.0 ± 8.1	6.4 ± 3.9^†^	6.0 ± 3.9*
TS Hip^G^*^T^	Pre	− 7.1 ± 8.5	− 7.9 ± 6.0	− 2.0 ± 6.0	− 3.4 ± 8.9
	Post	− 9.2 ± 6.0	− 6.5 ± 5.8	− 11.5 ± 2.4^†^	− 6.6 ± 5.4^†^
PS Ankle	Pre	3.1 ± 5.7	5.1 ± 6.2	0.8 ± 4.5	0.1 ± 5.4
	Post	2.0 ± 4.7	3.8 ± 6.7	0.3 ± 6.6	− 1.0 ± 7.9
PS Knee^L^	Pre	17.0 ± 9.6	18.3 ± 9.4^‡^	14.6 ± 11.7	33.2 ± 7.3^‡^
	Post	17.2 ± 11.7	18.9 ± 5.5^‡^	19.2 ± 9.8	31.1 ± 4.2^‡^
OS Hip	Pre	− 1.3 ± 9.7	2.2 ± 7.0	2.4 ± 3.8	6.1 ± 7.9
	Post	5.1 ± 8.5	3.8 ± 4.5	2.2 ± 5.3	1.6 ± 5.2
IS Ankle^L^	Pre	− 1.6 ± 0.9	− 15.2 ± 11.7^‡^	− 1.6 ± 1.2	− 15.2 ± 6.5^‡^
	Post	− 2.9 ± 2.0	− 15.5 ± 7.9^‡^	− 1.7 ± 1.9	− 17.8 ± 6.7^‡^
IS Knee^L^	Pre	37.0 ± 20.6	38.9 ± 14.3^‡^	30.5 ± 19.3	54.2 ± 8.2^‡^
	Post	40.1 ± 17.2	39.3 ± 10.3^‡^	41.9 ± 16.1	51.4 ± 7.1^‡^
IS Hip^L^	Pre	5.7 ± 4.0	5.9 ± 3.6^‡^	8.6 ± 8.2	21.2 ± 6.5^‡^
	Post	7.3 ± 4.1	13.5 ± 2.3^‡^	14.0 ± 9.6	14.4 ± 7.7^‡^
MS Ankle^L^	Pre	− 1.3 ± 1.1	4.9 ± 1.9^‡^	− 1.9 ± 1.0	5.0 ± 3.8^‡^
	Post	− 2.5 ± 2.0	3.6 ± 3.0^‡^	− 1.5 ± 1.4	7.4 ± 4.7^‡^
MS Knee^L^*^T^	Pre	10.2 ± 6.2	17.8 ± 10.4^‡^	10.2 ± 9.1	21.0 ± 10.5^‡^
	Post	20.3 ± 11.3	12.3 ± 6.4	22.4 ± 9.7^†^	24.5 ± 10.3
MS Hip^L^	Pre	10.5 ± 6.7	12.0 ± 4.3^‡^	15.4 ± 11.7	22.9 ± 7.3^‡^
	Post	11.9 ± 5.4	20.1 ± 8.7^‡^	14.9 ± 10.9	22.3 ± 7.1^‡^
Ts Ankle^L^	Pre	− 1.6 ± 1.2	5.0 ± 3.2^‡^	− 2.0 ± 1.1	4.7 ± 1.8^‡^
	Post	− 2.3 ± 1.9	2.1 ± 2.8^‡^	− 1.3 ± 1.2	7.2 ± 5.2^‡^
Ts Knee^L; T^	Pre	2.2 ± 2.8	5.7 ± 5.1^‡^	1.9 ± 0.7	8.8 ± 8.1^‡^
	Post	4.1 ± 7.0	2.9 ± 2.0^‡†^	1.7 ± 1.7^†^	3.1 ± 5.5^‡†^
Ts Hip^L^	Pre	12.2 ± 6.1	13.0 ± 3.7^‡^	16.1 ± 10.1	22.4 ± 4.9^‡^
	Post	14.2 ± 5.4	19.8 ± 7.7^‡^	16.6 ± 9.4	21.9 ± 7.1^‡^

Phases of gait cycle are abbreviated as: IC (0–2% CG)—Initial contact; LR (0–10% CG)—Loading response; MS (10–30% CG) – Mid Stance; TS (30–50% CG) – Terminal Stance; PS (50–60% CG)—Pre− swing; IS (60–73% CG)—Initial swing; MS (73–87% CG) – Mid swing; Ts (87–100% CG) – Terminal swing. Ankle dorsiflexion ( +) and plantar flexion (-); Knee flexion ( +) and extension (-); Hip flexion ( +) and extension (-).^G^ Main effect of group (*P* < 0.05); ^L^ Main effect of Limb (*P* < 0.05); ^T^ Main effect of time (*P* < 0.05); ^G^*^L^ Group*Limb interaction (*P* < 0.05); ^G^*^T^ Group*time interaction (*P* < 0.05); ^L^*^T^ Limb*time interaction (*P* < 0.05); ^G^*^L^*^T^ Group*Limb*time interaction (*P* < 0.05); * Difference between groups (*P* < 0.05); † Pre time difference (*P* < 0.05); ‡ Amputated limb difference.

**Figure 3:**
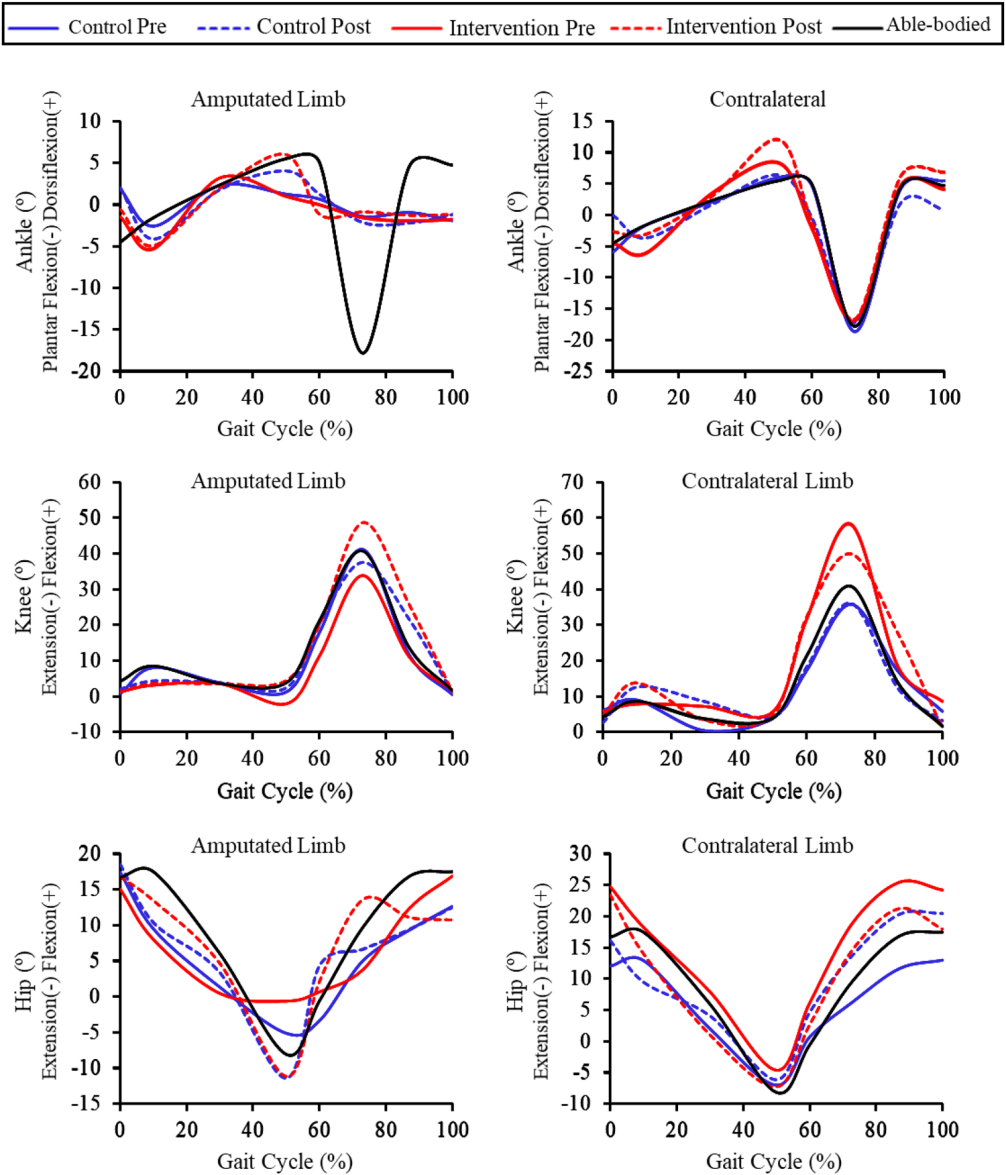
Ankle, knee and hip joint angular variation in the sagittal plane during different phases of gait cycle, in each group of individuals with amputation pre- and post-intervention. Able-bodied group parameter from their dominant limb is added for reference.

The magnitude of the effects can be seen in Table 4. Ankle, knee and hip joint angular variation during different gait phases pre- and post-intervention can be visualized in the representation of the gait cycle in Fig. 3.

**Table 4:** Analysis of the magnitude of the intervention effects on ankle, knee, and hip joint angles (º) in the sagittal plane during different phases of gait cycle.

Variables	Amputated limb				Contralateral limb			
	MD	ES	IC 95%		MD	ES	IC 95%	
*Control (n* = *7)*								
IC Ankle	− 1.36	− 0.30	− 1.25	0.66	3.87	0.86	0.02	1.70
IC Knee	0.60	0.10	− 0.75	0.96	0.31	0.05	− 0.82	0.91
IC Hip	3.74	0.64	− 0.18	1.45	6.38	0.78	− 0.05	1.61
LR Ankle	− 0.52	− 0.10	− 1.00	0.80	− 1.20	− 0.39	− 1.37	0.59
LR Knee	− 2.28	− 0.43	− 1.43	0.56	4.88	1.16	0.26	2.06
LR Hip	0.39	0.09	− 0.77	0.95	4.94	0.49	− 0.33	1.30
MS Ankle	− 0.20	− 0.09	− 0.98	0.81	− 0.47	− 0.24	− 1.18	0.69
MS Knee	− 0.11	− 0.03	− 0.91	0.86	4.06	0.69	− 0.13	1.50
MS Hip	2.21	0.96	0.11	1.82	3.27	0.53	− 0.28	1.35
TS Ankle	2.52	0.72	− 0.10	1.55	− 0.46	− 0.09	− 0.98	0.81
TS Knee	0.74	0.15	− 0.70	1.00	− 0.01	0.00	− 0.88	0.88
TS Hip	− 2.09	− 0.27	− 1.21	0.68	1.41	0.22	− 0.61	1.06
PS Ankle	− 1.04	− 0.19	− 1.11	0.74	− 1.31	− 0.19	− 1.11	0.73
PS Knee	0.21	0.02	− 0.85	0.89	0.61	0.07	− 0.79	0.94
PS Hip	6.43	0.66	− 0.16	1.48	1.62	0.26	− 0.57	1.09
IS Ankle	− 1.27	− 0.77	− 1.88	0.34	− 0.29	− 0.03	− 0.91	0.86
IS Knee	3.12	0.15	− 0.69	1.00	0.38	0.03	− 0.84	0.90
IS Hip	1.58	0.37	− 0.45	1.19	7.62	2.33	0.94	3.72
MS Ankle	− 1.16	− 0.67	− 1.74	0.41	− 1.31	− 0.49	− 1.50	0.53
MS Knee	10.11	1.04	0.17	1.91	− 5.48	− 0.59	− 1.64	0.45
MS Hip	1.34	0.20	− 0.63	1.04	8.07	1.09	0.21	1.98
Ts Ankle	− 0.69	− 0.41	− 1.39	0.58	− 2.99	− 0.94	− 2.11	0.24
Ts Knee	1.92	0.34	− 0.49	1.16	− 2.84	− 0.69	− 1.77	0.39
Ts Hip	1.93	0.31	− 0.51	1.14	6.77	1.04	0.17	1.92
*Intervention (n* = *7)*								
IC Ankle	0.39	0.13	− 0.72	0.98	0.25	0.05	− 0.81	0.92
IC Knee	− 0.45	− 0.36	− 1.33	0.61	− 1.61	− 0.26	− 1.21	0.68
IC Hip	0.95	0.13	− 0.72	0.98	− 0.09	− 0.01	− 0.89	0.87
LR Ankle	0.67	0.11	− 0.74	0.97	0.51	0.10	− 0.75	0.96
LR Knee	0.70	0.09	− 0.76	0.95	1.24	0.18	− 0.67	1.02
LR Hip	3.46	1.01	0.14	1.87	− 2.91	− 0.53	− 1.56	0.50
MS Ankle	− 0.93	− 0.26	− 1.20	0.68	− 0.36	− 0.11	− 1.01	0.79
MS Knee	1.02	0.16	− 0.69	1.01	− 1.71	− 0.31	− 1.26	0.65
MS Hip	2.16	0.57	− 0.25	1.38	− 5.93	− 1.37	− 2.71	− 0.03
TS Ankle	1.32	0.39	− 0.43	1.21	2.83	0.53	− 0.29	1.34
TS Knee	4.38	0.70	− 0.12	1.52	0.40	0.10	− 0.75	0.96
TS Hip	− 9.47	− 1.93	− 3.51	− 0.35	− 3.20	− 0.41	− 1.40	0.58
PS Ankle	− 0.48	− 0.08	− 0.97	0.82	− 1.06	− 0.15	− 1.06	0.77
PS Knee	4.61	0.40	− 0.42	1.22	− 2.10	− 0.33	− 1.29	0.63
PS Hip	− 0.15	− 0.03	− 0.91	0.85	− 4.43	− 0.62	− 1.68	0.44
IS Ankle	− 0.08	− 0.04	− 0.93	0.84	− 2.60	− 0.37	− 1.35	0.61
IS Knee	11,41	0.60	− 0.21	1.41	− 2.74	− 0.33	− 1.30	0.63
IS Hip	5.37	0.56	− 0.25	1.38	− 6.78	− 0.89	− 2.05	0.26
MS Ankle	0.39	0.29	− 0.54	1.12	2.39	0.52	− 0.29	1.34
MS Knee	12.12	1.20	0.29	2.12	3.44	0.31	− 0.52	1.14
MS Hip	− 0.46	− 0.04	− 0.92	0.85	− 0.60	− 0.08	− 0.97	0.82
Ts Ankle	0.70	0.58	− 0.23	1.40	2.51	0.61	− 0.21	1.42
Ts Knee	− 0.16	− 0.11	− 1.02	0.79	− 5.69	− 0.77	− 1.87	0.34
Ts Hip	0.52	0.05	− 0.82	0.92	− 0.47	− 0.07	− 0.96	0.82

MD: Mean difference; ES: Effect size; 95% CI: 95% confidence interval. Phases of the gait cycle are abbreviated as: IC (0–2% GC)—Initial contact; LR (0–10% GC)—Loading response; MS (10–30% GC) – Mid stance; TS (30–50% GC) – Terminal stance; PS (50–60% GC)—Pre-swing; IS (60–73% GC)—Initial swing; MS (73–87% GC) – Mid swing; Ts (87–100% GC) – Terminal swing. Dorsiflexion ( +) and plantar flexion of the ankle (-); Flexion ( +) and extension of the knee (-); Flexion ( +) and extension (-) of the hip.

### Kinetics: intervention vs. control

In the comparison between limbs and groups, for the ankle joint, lower plantar flexion moment during initial contact, and lower dorsiflexion moment during mid stance in the contralateral limb were found in the intervention group.

For the knee joint, between limbs, higher flexion moment during loading response, mid stance and terminal stance was found in the contralateral limb; between time points, lower flexion moment during initial contact was found post-intervention.

For the hip joint, between limbs, lower flexion moment during pre-swing was found in the contralateral limb; between groups, lower extension moment during initial contact was found in the intervention group; pre-intervention between limbs, higher extension moment during initial was found in the contralateral limb; post-intervention between groups, higher flexion moment during mid stance was found in the intervention group; within the control group between time points, higher extension moment was found during mid stance (Table 5).

**Table 5:** Ankle, knee, and hip joint moment (Nm/Kg) in the sagittal plane during different phases of gait cycle, in amputees pre- and post-intervention.

Variables		Control		Intervention	
		Amputated limb	Contralateral limb	Amputated limb	Contralateral limb
IC Ankle^*G*^	Pre	− 0.11 ± 0.07	− 0.13 ± 0.11	− 0.10 ± 0.03*	− 0.09 ± 0.09*
	Post	− 0.32 ± 0.51	− 0.10 ± 0.08	− 0.06 ± 0.02*	− 0.07 ± 0.06*
IC Knee^*T*^	Pre	− 0.15 ± 0.11	− 0.17 ± 0.22	− 0.16 ± 0.18	− 0.10 ± 0.40
	Post	− 0.07 ± 0.25^†^	0.13 ± 0.18^†^	− 0.09 ± 0.06^†^	0.00 ± 0.21^†^
IC Hip^G;L^*^T^	Pre	0.28 ± 0.39	0.58 ± 0.50^‡^	0.15 ± 0.09*	0.57 ± 0.30*^‡^
	Post	0.64 ± 1.04	0.40 ± 0.26	0.23 ± 0.19*	0.29 ± 0.24*
LR Ankle	Pre	− 0.56 ± 0.59	− 0.32 ± 0.23	− 0.14 ± 0.12	− 0.23 ± 0.31
	Post	− 0.24 ± 0.80	− 0.05 ± 0.25	− 0.19 ± 0.16	0.07 ± 0.37
LR Knee^*L*^	Pre	− 0.10 ± 0.51	0.30 ± 0.26^‡^	0.03 ± 0.14	0.26 ± 0.25^‡^
	Post	− 0.25 ± 0.81	0.21 ± 0.18^‡^	0.01 ± 0.25	0.34 ± 0.34^‡^
LR Hip	Pre	0.36 ± 0.40	0.73 ± 0.36	0.37 ± 0.39	0.72 ± 0.36
	Post	0.63 ± 0.66	0.40 ± 0.39	0.34 ± 0.30	0.34 ± 0.24
MS Ankle^*L*^	Pre	1.71 ± 2.03	0.51 ± 0.36^‡^	3.38 ± 3.84	0.89 ± 1.38^‡^
	Post	1.98 ± 2.91	0.62 ± 0.68^‡^	3.21 ± 3.71	0.95 ± 1.24^‡^
MS Knee	Pre	− 1.58 ± 2.91	0.49 ± 0.41^‡^	− 1.07 ± 5.92	1.20 ± 2.54^‡^
	Post	0.89 ± 4.15	0.78 ± 1.17^‡^	− 0.77 ± 6.06	1.09 ± 1.69^‡^
MS Hip^G^*^L^	Pre	− 2.07 ± 3.49	− 0.61 ± 0.32	− 4.21 ± 5.14	− 1.44 ± 3.01
	Post	2.67 ± 3.70^†^	0.90 ± 1.30^†^	− 4.24 ± 5.44*	− 0.63 ± 2.49*
TS Ankle	Pre	1.49 ± 2.49	1.63 ± 1.54	3.69 ± 4.31	1.44 ± 1.22
	Post	2.14 ± 2.94	0.92 ± 0.80	3.58 ± 4.09	1.64 ± 1.39
TS Knee^*L*^	Pre	− 1.58 ± 3.55	0.52 ± 3.14^‡^	− 4.70 ± 5.53	0.69 ± 2.92^‡^
	Post	0.49 ± 5.07^†^	1.02 ± 1.45^‡^	− 4.66 ± 5.68^†^	1.22 ± 2.67^‡†^
TS Hip	Pre	− 2.03 ± 4.67	− 0.46 ± 0.38	− 5.94 ± 6.98	− 0.13 ± 0.26
	Post	− 0.26 ± 6.25	1.17 ± 3.61	− 5.87 ± 7.31	1.17 ± 3.61
PS Ankle	Pre	1.11 ± 2.68	1.00 ± 1.81	3.66 ± 4.48	0.94 ± 1.68
	Post	0.63 ± 1.41	0.47 ± 0.79	3.49 ± 4.52	1.29 ± 1.76
PS Knee	Pre	1.64 ± 4.28	1.35 ± 3.20	5.44 ± 6.99	1.45 ± 3.54
	Post	− 1.18 ± 3.23	1.02 ± 2.04	5.56 ± 7.17	1.62 ± 3.71
PS Hip^*L*^	Pre	− 2.27 ± 5.82	0.85 ± 3.28^‡^	− 7.04 ± 8.34	1.03 ± 3.49^‡^
	Post	− 2.01 ± 4.88	1.06 ± 2.11^‡^	− 6.78 ± 8.51	1.64 ± 4.48^‡^

Phases of the gait cycle are abbreviated as: IC (0–2% GC)—Initial contact; LR (0–10% GC)—Loading response; MS (10–30% GC) – Mid stance; TS (30–50% GC) – Terminal stance; PS (50–60% GC)—Pre-swing. Dorsiflexion ( +) and plantar flexion of the ankle (-); Flexion ( +) and extension of the knee (-); Flexion ( +) and extension (-) of the hip. ^*G*^ Main effect of group (*P* < 0.05); ^*L*^ Main effect of limb (*P* < 0.05); ^*T*^ Main effect of time (*P* < 0.05); ^G^*^L^ Group*limb interaction (*P* < 0.05); ^L^*^T^ Limb*time interaction (*P* < 0.05); * Between-group difference (*P* < 0.05); † Pre-time difference (*P* < 0.05); ‡ Amputated limb difference.

**Figure 4:**
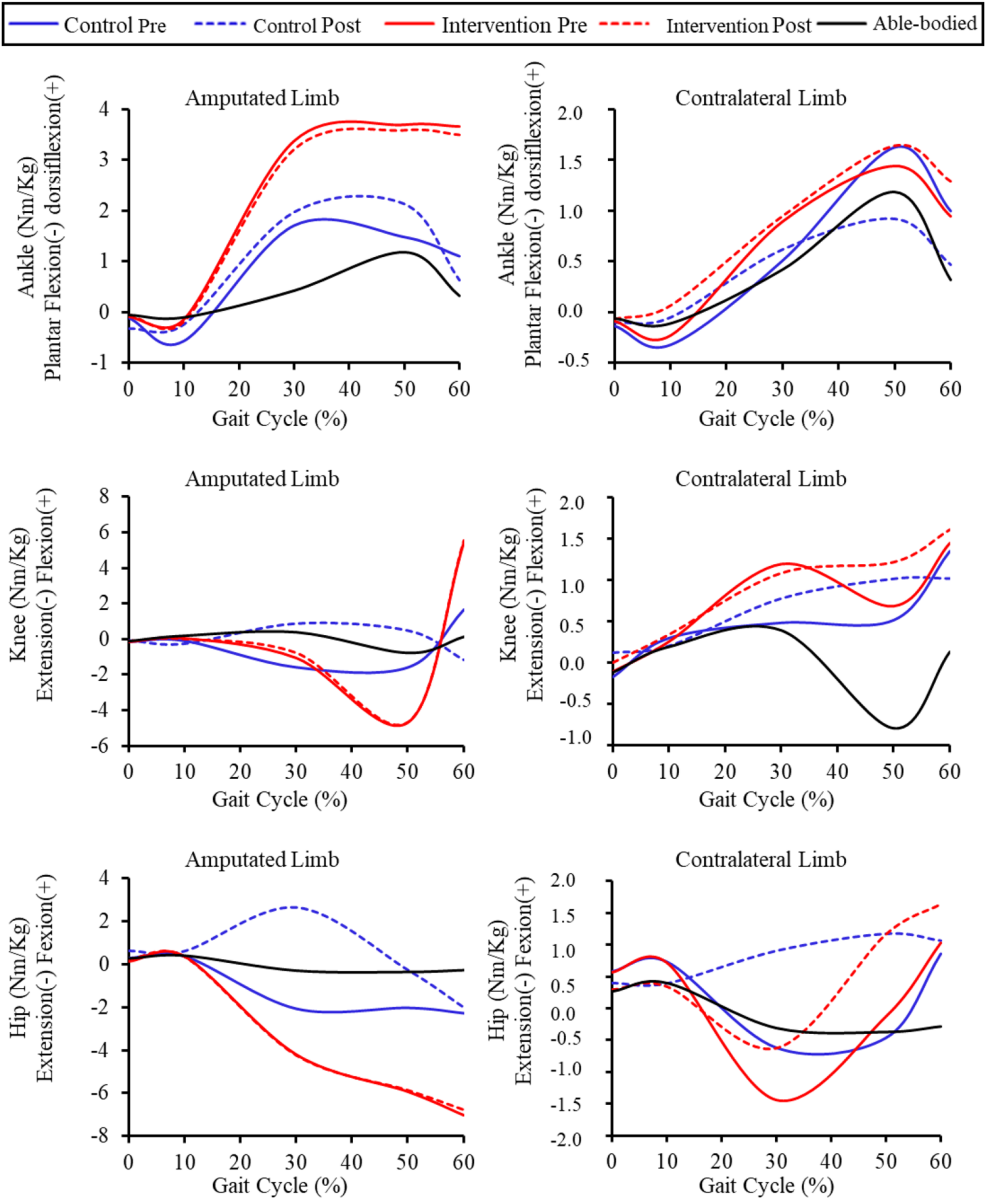
Ankle, knee and hip joint moment variation in the sagittal plane during gait cycle, in each group with amputation pre- and post-intervention. Able-bodied group parameter from their dominant limb is added for reference.

The magnitude of the effects for different groups, limbs and time points can be seen in Table 6. Average joint moments can be visualized in the representation of the gait cycle in Fig. 4.

**Table 6:** Analysis of the magnitude of the intervention effects on ankle, knee, and hip joint moments (Nm/Kg) in the sagittal plane during stance phase.

Variables	Amputated limb				Contralateral limb			
	MD	ES	CI 95%		MD	ES	CI 95%	
*Control (n* = *7)*								
IC Ankle	− 0.22	− 0.56	− 1.60	0.47	0,03	0.33	− 0.49	1.15
IC Knee	0.07	0.36	− 0.46	1.18	0.29	1.35	0.39	2.32
IC Hip	0.36	0.42	− 0.39	1.24	− 0.18	− 0.42	− 1.41	0.57
LR Ankle	0.32	0.43	− 0.39	1.24	0.27	1.06	0.18	1.93
LR Knee	− 0.15	− 0.21	− 1.13	0.72	− 0.10	− 0.40	− 1.38	0.59
LR Hip	0.26	0.45	− 0.36	1.27	− 0.33	− 0.83	− 1.96	0.30
MS Ankle	0.26	0.10	− 0.76	0.96	0.11	0.19	− 0.65	1.03
MS Knee	2.47	0.65	− 0.17	1.46	0.29	0.31	− 0.51	1.14
MS Hip	4.74	1.23	0.31	2.16	1.52	1.60	0.55	2.65
TS Ankle	0.65	0.22	− 0.61	1.06	− 0.70	− 0.53	− 1.56	0.49
TS Knee	2.07	0.44	− 0.37	1.26	0.50	0.19	− 0.65	1.03
TS Hip	1.77	0.30	− 0.53	1.13	1.63	0.59	− 0.22	1.41
PS Ankle	− 0.48	− 0.21	− 1.14	0.72	− 0.53	− 0.35	− 1.32	0.62
PS Knee	− 2.82	− 0.70	− 1.78	0.39	− 0.33	− 0.12	− 1.03	0.78
PS Hip	0.26	0.05	− 0.82	0.91	0.21	0.07	− 0.79	0.93
*Intervention (n* = *7)*								
IC Ankle	0.04	1.20	0.29	2.12	0.02	0.33	− 0.49	1.15
IC Knee	0.07	0.47	− 0.34	1.29	0.10	0.32	− 0.51	1.14
IC Hip	0.08	0.57	− 0.25	1.38	− 0.27	− 1.01	− 2.20	0.19
LR Ankle	− 0.04	− 0.32	− 1.28	0.64	0.30	0.88	0.04	1.72
LR Knee	− 0.02	− 0.11	− 1.01	0.79	0.08	0.27	− 0.56	1.10
LR Hip	− 0.04	− 0.10	− 1.01	0.80	− 0.37	− 1.22	− 2.51	0.06
MS Ankle	− 0.17	− 0.04	− 0.93	0.84	0.06	0.04	− 0.82	0.91
MS Knee	0.30	0.05	− 0.82	0.92	− 0.11	− 0.05	− 0.94	0.84
MS Hip	− 0.04	− 0.01	− 0.89	0.87	0.81	0.29	− 0.53	1.12
TS Ankle	− 0.11	− 0.03	− 0.91	0.86	0.20	0.15	− 0.69	1.00
TS Knee	0.03	0.01	− 0.87	0.88	0.53	0.19	− 0.65	1.03
TS Hip	0.07	0.01	− 0.86	0.88	1.30	0.51	− 0.31	1.32
PS Ankle	− 0.17	− 0.04	− 0.92	0.85	0.35	0.20	− 0.64	1.04
PS Knee	0.13	0.02	− 0.85	0.89	0.16	0.04	− 0.82	0.91
PS Hip	0.26	0.03	− 0.84	0.90	0.61	0.15	− 0.69	1.00

MD: Mean difference; ES: Effect size; 95% CI: 95% confidence interval. Phases of the gait cycle are abbreviated as: IC (0–2% GC)—Initial contact; LR (0–10% GC)—Loading response; MS (10–30% GC) – Mid stance; TS (30–50% GC) – Terminal stance; PS (50–60% GC)—Prerswing; Dorsiflexion ( +) and plantar flexion of the ankle (-); Flexion ( +) and extension of the knee (-); Flexion ( +) and extension (-) of the hip.

### Temporal-spatial: intervention vs. control

Both mean gait speed and cadence increased at the post-intervention compared to pre-intervention, while for stride length, both groups increased (Table 7). The interpretation of effect magnitude can be seen in Table 8.

**Table 7:** Temporal-spatial parameters of gait, in each amputee group pre- and post-intervention.

Variables		Groups	
		control (N = 7)	Intervention (N = 7)
Gait speed (m/s)^G^*^T^	Pre	0.51 ± 0.22	0.28 ± 0.15
	Post	0.51 ± 0.23	0.63 ± 0.21
Cadence (passo/min)^G^*^T^	Pre	72.9 ± 31.8	42.6 ± 18.7
	Post	73.1 ± 32.4	85.7 ± 29.7
*Step length (m)*			
*Amputated*	Pre	0.34 ± 0.05	0.32 ± 0.04
*Amputated*	Post	0.37 ± 0.07	0.39 ± 0.03
*Contralateral*	Pre	0.35 ± 0.06	0.33 ± 0.04
*Contralateral*	Post	0.38 ± 0.08	0.39 ± 0.04
*Stride length (m)*			
*Amputated*	Pre	0.69 ± 0.11	0.65 ± 0.08
*Amputated*	Post	0.75 ± 0.16	0.78 ± 0.06
*Contralateral*	Pre	0.69 ± 0.11	0.65 ± 0.08
*Contralateral*	Post	0.75 ± 0.16	0.78 ± 0.06
Stance phase %	Pre	47.8 ± 32.3	65.1 ± 22.8
	Post	64.5 ± 6.8	57.5 ± 16.7

^G^*^T^ Group*time interaction (*P* < 0.05); † Pre time difference (*P* < 0.05).

**Table 8:** Analysis of the magnitude of the intervention effects on temporal-spatial parameters of gait, in each amputee group.

Variables	Control (N = 7)				Intervention (N = 7)			
	MD	ES	CI 95%		MD	ES	CI 95%	
Gait speed (m/s)	0.00	0.01	− 0.86	0.89	0.35	1.83	0.68	2.98
Cadence (passo/min)	0.29	0.01	− 0.87	0.88	43.14	1.63	0.56	2.69
*Step length (m)*								
*Amputated*	0.04	0.54	− 0.27	1.35	0.07	1.92	0.73	3.11
*Contralateral*	0.03	0.41	− 0.40	1.23	0.05	1.17	0.26	2.07
*Stride length (m)*								
*Amputated*	0.06	0.41	− 0.41	1.23	0.13	1.73	0.62	2.84
*Contralateral*	0.06	0.40	− 0.42	1.22	0.13	1.71	0.61	2.81
Stance phase %	16.69	0.67	− 0.15	1.49	− 7.61	− 0.36	− 1.33	0,62

MD: Mean difference; ES: Effect size; 95% CI: 95% confidence interval.

## Discussion

The 16-week physiotherapy intervention protocol presented in this study demonstrated to be effective as the first stage of a post-amputation rehabilitation process. With regards to the magnitude of the intervention effect on temporal-spatial parameters, for the intervention group, the results demonstrated an increase in gait speed, cadence, and step length, with a large effect size, when compared to the control group. Stride length increased in both groups. However, the intervention group had a large effect size, while the control group had a moderate effect size (Table 8). From the combination of multiple gait parameters, it can be inferred that the intervention protocol promoted improvement in gait parameters, especially in temporal-spatial variables, and prosthesis adaptation. Despite the improvement in kinematic and kinetic parameters of gait, the study showed that both groups adopted adaptive gait strategies, presenting variability and asymmetry between limbs.

Previous studies showed that individuals who received intervention after amputation, not only tended to improve musculoskeletal resistance, gait speed, and the capacity to adjust to the prosthesis, but the intervention also reverberated in an increase of the survival rate by 1 year, when compared to people who did not receive any treatment^18–21^. Other studies, such as the present one, pointed out the adoption of adaptive patterns and the asymmetry between limbs, persisting during gait, also when sitting, getting up, and climbing stairs and ramps^22–24^. Although the origin of gait variability in individuals with amputation is not well defined, studies showed that it is related to multisystemic issues such as neuromotor control dysfunction, peripheral and central nervous system function, autonomic nervous system, and cardiac, musculoskeletal and psychological adaptation^25–27^.

In the stance phase, the main differences between intervention and control groups were in ankle (group and limb) and hip (group and time) joint angle during initial contact. For the ankle joint, the intervention group presented higher plantar flexion for both limbs, amputated and contralateral. For the hip joint, the intervention group presented higher flexion pre- and post-intervention. During mid stance, the hip showed a significant difference between interaction*group, limb and time, with a large effect size for the contralateral limb. In other words, there was a greater hip extension for contralateral limb post-intervention. During terminal stance, for the intervention group, there was an increase of knee flexion in the amputated limb, and greater hip extension in the amputated and contralateral limbs post-intervention. In the control group, there was a smaller hip extension and no difference between limbs or times. In the swing phase, the data did not present differences between the groups, only between limbs, as shown by a knee angle difference between limbs and times in the mid and terminal swing.

For the sagittal plane kinetic data, considered as propellants of the movement, the main difference observed by the study between intervention and control groups regarding moments, were for ankle plantarflexion moment during initial contact. The intervention group demonstrated a neutral tendency for ankle moment, for the amputated and contralateral limbs, and the control group demonstrated a tendency for a plantarflexion moment, mainly for the amputated limb. Differences between groups during initial contact were also for hip moment, showing a lower flexor tendency for the intervention when compared to control group. During mid stance, also in the hip segment, the intervention group presented a moment with extensor tendency, while the control group presented a moment with flexor tendency.

A previous study also investigating individuals with TT and TF amputation obtained similar adaptations at the hip joint to compensate for the loss of the ankle joint. Accordingly, differences at the knee joint were observed depending on amputation level, also similar to the findings of the present study^28^. Another study showed that the proposed rehabilitation exercise program promoted stability during gait phases, and the number of falls were reduced^29^. A study carried out with individuals with TT amputation showed positive results, with 66.7% improvement in functional mobility after undergoing a rehabilitation protocol^30^.

A limitation of this study was the small sample size; it prevented the separation of groups by level of amputation and cause of amputation. In addition, the different types and models of footwear wore by the participants during the process of adaptation to prosthesis, rehabilitation and gait assessments were also configured as possible limitations of the study.

In conclusion, this study showed that a low-cost and easily implementable physiotherapy intervention was relevant as the very first stage of a post-amputation rehabilitation procedure to aid in an individual’s adaptation to wearing prosthesis. There was improvement of kinetic and kinematic variables resulting in better gait performance by increasing speed, stride and step size. In addition, there was a decrease in stance contact time of the amputated limb, thus demonstrating a greater fluidity of gait after the intervention. However, asymmetry was observed between lower limb in both groups.

Additionally, it confirmed the importance of ensuring the continuity of rehabilitation and monitoring of individuals by a multidisciplinary team post-prosthesis fitting, mainly because this population is more susceptible to falls, requiring more neuromotor training to improve the performance of gait and daily activities.
